# Extra-Paramidal Side Effects Associated with Paroxetine

**Published:** 1992-12

**Authors:** Simon D. Nicholson

**Affiliations:** Psychiatric Department North Devon District Hospital Raleigh Park, Barnstaple EX31 4JR

## Abstract

A 29-year-old man admitted to a psychiatric unit was successfully treated for a paranoid psychosis with trifluoperazine. Following reduction of neuroleptic dosage and addition of paroxetine he developed severe extra-pyramidal side effects. These remitted after withdrawal of medication and did not recur following recommencement of trifluoperazine. This is the first report of such a side effect associated with paroxetine use in man.


### Extra Pyramidal Side Effects Associated with Paroxetine

**Simon D. Nicholson,**
**M.B., B.S., B.Sc., D.G.M., M.R.C.Psych.**
Psychiatric Department
North Devon District Hospital
Raleigh Park, Barnstaple EX31 4JR

### SUMMARY
A 29-year-old man admitted to a psychiatric unit was successfully treated for a paranoid psychosis with trifluoperazine. Following reduction of neuroleptic dosage and addition of paroxetine he developed severe extra-pyramidal side effects. These remitted after withdrawal of medication and did not recur following recommencement of trifluoperazine. This is the first report of such a side effect associated with paroxetine use in man.

### INTRODUCTION
Selective serotonin reuptake inhibitors (SSRI) are commonly used antidepressants with a reputation for safety and efficacy. A recent commentator[1] suggests they are particularly useful in cases of depression accompanying obesity or heart disease or if the patient is unable to tolerate tricyclics. They may be especially useful in the frail elderly and may have place in the treatment of obsessive compulsive disorder and bulimia nervosa. Despite this they are sometimes associated with adverse reactions of which an extra-pyramidal syndrome is one. The following is an account of such a syndrome associated with paroxetine.

### A CASE REPORT
A 29-year-old man, admitted to a psychiatric unit, complained of an unpleasant smell in his home and of a conspiracy against him by the Freemasons. Mental state examination revealed delusions, ideas of reference, formal thought disorder and low mood. There was no cognitive impairment and physical examination was unremarkable. The following investigations proved negative: urea and electrolytes, thyroid and liver function tests, urine drug screen, full blood count, serum viscosity and urine culture and sensitivity. A diagnosis of schizophreniform psychosis was made and the patient commenced on trifluoperazine 15 mg per day. Slight extra-pyramidal side effects developed and procyclidine hydrochloride 5 mg bd was added. Over 3-4 weeks the patient's psychotic symptoms receded and his mood improved.

Three weeks following discharge the trifluoperazine was reduced to 10 mg per day. Soon afterwards the patient returned to work but became anxious and was prescribed paroxetine 20 mg per day. Over the next 3-4 days he developed extra-pyramidal symptoms which necessitated a second admission. He was unable to walk unaided, suffered from cogwheel rigidity affecting his upper limbs and had parkinsonian facies with drooling. He was not psychotic. Trifluoperazine and paroxetine were stopped but procyclidine hydrochloride continued at 5 mg tds. Over 7 days the patient's motor symptoms abated and he was discharged on no medication.

Less than a week later a return of the patient's psychotic symptoms prompted his GP to prescribe trifluoperazine 5 mg per day and procyclidine hydrochloride 5 mg per day. The antipsychotic medication was later increased to 10 mg per day and the patient continues to be seen in the out-patient clinic where he is troubled by mild psychotic symptoms but no extra-pyramidal side effects.

### DISCUSSION
The onset of rigidity soon after commencing anti-depressant therapy and the absence of marked extra-pyramidal symptoms associated with trifluoperazine alone suggest that paroxetine was a necessary though possibly not sufficient cause of this patient's parkinsonism.

There are reports of extra-pyramidal symptoms arising from the use of other SSRIs either alone or in combination with neuroleptics. Meltzer et al[2] report a 25-year-old man who developed torticollis, jaw rigidity, cogwheel rigidity and abnormality of gait after taking fluoxetine. Tate[3] reports a 39 year-old woman who developed extra-pyramidal side effects whilst taking haloperidol and fluoxetine. She had been taking haloperidol for 2 years with only mild extra-pyramidal symptoms. Bouchard et al[4] describe a number of patients receiving fluoxetine who developed parkinsonian symptoms. They also refer to unpublished cases of patients with idiopathic Parkinson's disease reacting adversely to the same drug.

Paroxetine is 95% bound to plasma proteins[5] and a possible mechanism of the reported effect is displacement of neuroleptic from its binding sites on plasma proteins. However, it is possible that SSRIs exert an indirect inhibitory effect upon the dopaminergic cells of the substantia nigra and other regions within the CNS. Meltzer et al[2] found CSF homovanillic acid levels to be decreased in their patient on fluoxetine compared with pre-treatment levels and serum prolactin increased within 24 hours of starting the drug. Tryptophan and 5 hydroxtryptophan can worsen extra-pyramidal symptoms in Parkinson's disease[6, 7] and electrical stimulation of serotonergic neurones in the median raphe inhibit the dopaminenergic neurones of the substantia migra as does direct application of serotonin to the latter[8]. In addition it is possible that fluoxetine can inhibit the synthesis of Dopamine in the central nervous system[9].

The above case report is surprising since an association between paroxetine and extra-pyramidal symptoms has never been witnessed in man[10]. In addition paroxetine does not induce catalepsy in the rat[11] and there is no evidence from limited studies that it reduces central dopamine synthesis[12].

Clearly the clinical phenomena discussed above are rare and it is likely they are caused in most cases by disturbance of a nigrostriatal pathway which is already impaired through either Parkinson's disease or neuroleptic medication. This and previous case reports suggest caution in using SSRIs in patients who are already showing signs of nigrostriatal dysfunction. In addition it supports a growing body of evidence which suggests that -hydroxytryptamine has an important modulatory role with respect to central dopainergic pathways.

### REFERENCES
1. Edwards J. G. (1992). Selective serotonin re-uptake inhibitors: a modest though welcome advance in the treatment of depression. *British Medical Journal*: **6043**: Vol 304, 1644-1645.
2. Meltzer H. Y., Young M., Metz J., Fang V. S., Schyre P. M., Anora R. C. (1979). Extra pyramidal side effects and increased serum prolactin following fluoxetine, a new antidepressant. *Journal of Neural Transmission*: **45**: 165-175.
3. Tate J. (1989). Extra-pyramidal symptoms in a patient taking haloperidol and fluoxetine. *American Journal of Psychiatry*: **146**: 399-400.
4. Bouchard R., Parcher E. and Vincent P. (1989). Flouxetine and extrapyramidal side effects. *American Journal of Psychiatry*: **146**: 1352-1353.
5. Kaye C. M., Haddock R. E., Langley P. F., Mellows G., Tasker T. C. G., Zussman B. D. and Greb W. H. (1989). A review of the metabolism and pharmacokinetics of paroxetine in man. *Acta Psychiatrica Scandinavia*: **80 (supp. 350)**: 60-75.

6. Hall C. D., Weiss E. A., Morris C. E., Prange A. J. (1972). Rapid deterioration in patients with parkinsonism following tryptophan-pyridoxine administration. *Neurology*: **22**: 231-237.
7. Chase T. N., Ng L. K. Y., Watonabe A M. (1972). Parkinson's Modification by 5-hydroxytryptophan. *Neurology*: **22**: 479-484.
8. Dray A., Gonye T. J. Oakley N. R., Tanner T. (1976). Evidence for the existence of a raphe projection to the substantia nigra in rat. *Brain research*: **113**: 45-57.
9. Baldessarini R. J., Marsh E. (1990). Flouxetine and side effects. *Archives of general psychiatry*: **47**: 191-192.
10. Korsgaard S., Gerlack J. and Christensson E. (1985). Behavioural aspects of serotonin-dopamine interaction in the monkey. *European journal of pharmacology*: **118**: 245-252.
11. Johnson A. M., in *Perspectives in Psychiatry Vol 1: Selective Serotonin Re-uptake Inhibitors*. Eds: Feighner J P, Boyer W F. Page 58. Published by John Wiley & Sons (1991).
12. Johnson A. M., in *Perspectives in Psychiatry Vol 1: Selective Serotonin Re-uptake Inhibitors*. Eds; Feighner J. P., Boyer W. F. Page 58. Published by John Wiley & Sons (1991).

